# A mathematical model for simulating the phase-based transmissibility of a novel coronavirus

**DOI:** 10.1186/s40249-020-00640-3

**Published:** 2020-02-28

**Authors:** Tian-Mu Chen, Jia Rui, Qiu-Peng Wang, Ze-Yu Zhao, Jing-An Cui, Ling Yin

**Affiliations:** 1 State Key Laboratory of Molecular Vaccinology and Molecular Diagnostics, School of Public Health, Xiamen University, 4221-117 South Xiang’an Road, Xiang’an District, Xiamen, Fujian Province People’s Republic of China; 2 Department of Mathematics, School of Science, Beijing University of Civil Engineering and Architecture, Beijing, People’s Republic of China; 3 Shenzhen Institutes of Advanced Technology, Chinese Academy of Sciences, Shenzhen, Guangdong Province People’s Republic of China

**Keywords:** Novel coronavirus, Mathematical model, Basic reproduction number, Next generation matrix, Transmissibility

## Abstract

**Background:**

As reported by the World Health Organization, a novel coronavirus (2019-nCoV) was identified as the causative virus of Wuhan pneumonia of unknown etiology by Chinese authorities on 7 January, 2020. The virus was named as severe acute respiratory syndrome coronavirus 2 (SARS-CoV-2) by International Committee on Taxonomy of Viruses on 11 February, 2020. This study aimed to develop a mathematical model for calculating the transmissibility of the virus.

**Methods:**

In this study, we developed a Bats-Hosts-Reservoir-People transmission network model for simulating the potential transmission from the infection source (probably be bats) to the human infection. Since the Bats-Hosts-Reservoir network was hard to explore clearly and public concerns were focusing on the transmission from Huanan Seafood Wholesale Market (reservoir) to people, we simplified the model as Reservoir-People (RP) transmission network model. The next generation matrix approach was adopted to calculate the basic reproduction number (*R*_0_) from the RP model to assess the transmissibility of the SARS-CoV-2.

**Results:**

The value of *R*_0_ was estimated of 2.30 from reservoir to person and 3.58 from person to person which means that the expected number of secondary infections that result from introducing a single infected individual into an otherwise susceptible population was 3.58.

**Conclusions:**

Our model showed that the transmissibility of SARS-CoV-2 was higher than the Middle East respiratory syndrome in the Middle East countries, similar to severe acute respiratory syndrome, but lower than MERS in the Republic of Korea.

## Background

On 31 December 2019, the World Health Organization (WHO) China Country Office was informed of cases of pneumonia of unknown etiology (unknown cause) detected in Wuhan City, Hubei Province of China, and WHO reported that a novel coronavirus (2019-nCoV), which was named as severe acute respiratory syndrome coronavirus 2 (SARS-CoV-2) by International Committee on Taxonomy of Viruses on 11 February, 2020, was identified as the causative virus by Chinese authorities on 7 January [1]. It is reported that the virus might be bat origin [2], and the transmission of the virus might related to a seafood market (Huanan Seafood Wholesale Market) exposure [3, 4]. The genetic features and some clinical findings of the infection have been reported recently [4–6]. Potentials for international spread via commercial air travel had been assessed [7]. Public health concerns are being paid globally on how many people are infected and suspected.

Therefore, it is urgent to develop a mathematical model to estimate the transmissibility and dynamic of the transmission of the virus. There were several researches focusing on mathematical modelling [3, 8]. These researches focused on calculating the basic reproduction number (*R*_0_) by using the serial intervals and intrinsic growth rate [3, 9, 10], or using ordinary differential equations and Markov Chain Monte Carlo methods [8]. However, the bat origin and the transmission route form the seafood market to people were not considered in the published models.

In this study, we developed a Bats-Hosts-Reservoir-People (BHRP) transmission network model for simulating the potential transmission from the infection source (probably be bats) to the human infection. Since the Bats-Hosts-Reservoir network was hard to explore clearly and public concerns were focusing on the transmission from Huanan Seafood Wholesale Market (reservoir) to people, we simplified the model as Reservoir-People (RP) transmission network model, and *R*_0_ was calculated based on the RP model to assess the transmissibility of the SARS-CoV-2.

## Methods

### Data source

The reported cases of SARS-CoV-2, which have been named as COVID-19, were collected for the modelling study from a published literature [3]. As reported by Li et al. [3], the onset date of the first case was on 7 December, 2020, and the seafood market was closed on 1 January, 2020 [11]. The epidemic curve from 7 December, 2019 to 1 January, 2020 was collected for our study, and the simulation time step was 1 day.

### Simulation methods and statistical analysis

Berkeley Madonna 8.3.18 (developed by Robert Macey and George Oster of the University of California at Berkeley. Copyright©1993–2001 Robert I. Macey & George F. Oster) was employed for the curve fitting. The fourth-order Runge–Kutta method, with tolerance set at 0.001, was used to perform curve fitting. While the curve fitting is in progress, Berkeley Madonna displays the root mean square deviation between the data and best run so far. The coefficient of determination (*R*^2^) was employed to assess the goodness-of-fit. SPSS 13.0 (IBM Corp., Armonk, NY, USA) was employed to calculate the *R*^2^.

### The Bats-Hosts-Reservoir-People (BHRP) transmission network model

**Fig. 1:**
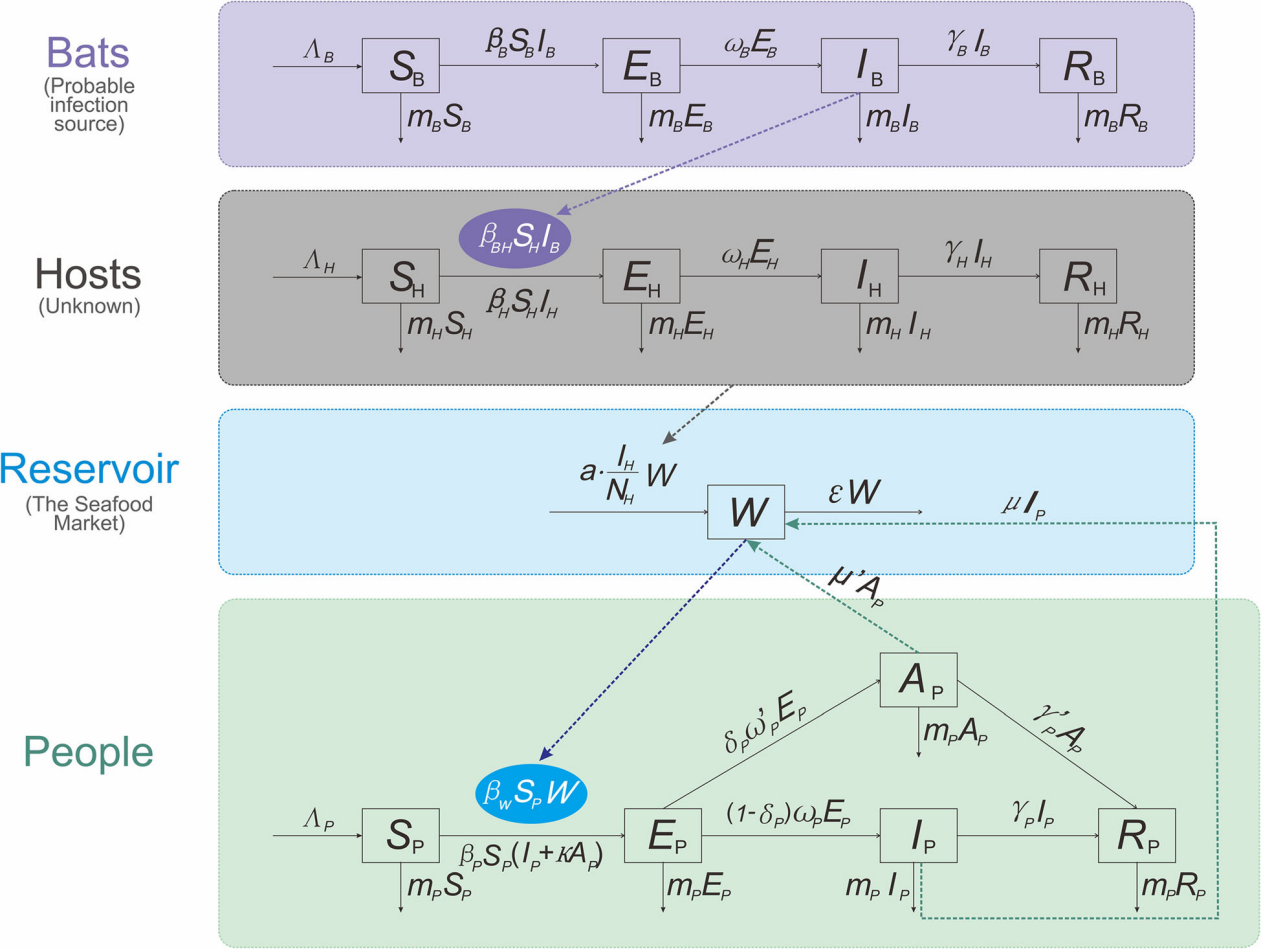
Flowchart of the Bats-Hosts-Reservoir-People transmission network model

The BHRP transmission network model was posted to bioRxiv on 19 January, 2020 [12]. We assumed that the virus transmitted among the bats, and then transmitted to unknown hosts (probably some wild animals). The hosts were hunted and sent to the seafood market which was defined as the reservoir of the virus. People exposed to the market got the risks of the infection (Fig. 1). The BHRP transmission network model was based on the following assumptions or facts:

- The bats were divided into four compartments: susceptible bats (*S*_*B*_), exposed bats (*E*_*B*_), infected bats (*I*_*B*_), and removed bats (*R*_*B*_). The birth rate and death rate of bats were defined as *n*_*B*_ and *m*_*B*_. In this model, we set *Ʌ*_*B*_ *= n*_*B*_ × *N*_*B*_ as the number of the newborn bats where *N*_*B*_ refer to the total number of bats. The incubation period of bat infection was defined as 1/*ω*_*B*_ and the infectious period of bat infection was defined as 1/*γ*_*B*_. The *S*_*B*_ will be infected through sufficient contact with *I*_*B*_, and the transmission rate was defined as *β*_*B*_.
- The hosts were also divided into four compartments: susceptible hosts (*S*_*H*_), exposed hosts (*E*_*H*_), infected hosts (*I*_*H*_), and removed hosts (*R*_*H*_). The birth rate and death rate of hosts were defined as *n*_*H*_ and *m*_*H*_. In this model, we set *Ʌ*_*H*_ *= n*_*H*_ × *N*_*H*_ where *N*_*H*_ refer to the total number of hosts. The incubation period of host infection was defined as 1/*ω*_*H*_ and the infectious period of host infection was defined as 1/*γ*_*H*_. The *S*_*H*_ will be infected through sufficient contact with *I*_*B*_ and *I*_*H*_, and the transmission rates were defined as *β*_*BH*_ and *β*_*H*_, respectively.
- The SARS-CoV-2 in reservoir (the seafood market) was denoted as *W*. We assumed that the retail purchases rate of the hosts in the market was *a*, and that the prevalence of SARS-CoV-2 in the purchases was *I*_*H*_/*N*_*H*_, therefore, the rate of the SARS-CoV-2 in *W* imported form the hosts was *aWI*_*H*_/*N*_*H*_ where *N*_*H*_ was the total number of hosts. We also assumed that symptomatic infected people and asymptomatic infected people could export the virus into *W* with the rate of μ_P_ and *μ’*_*P*_, although this assumption might occur in a low probability. The virus in *W* will subsequently leave the *W* compartment at a rate of *εW*, where 1/*ε* is the lifetime of the virus.
- The people were divided into five compartments: susceptible people (*S*_*P*_), exposed people (*E*_*P*_), symptomatic infected people (*I*_*P*_), asymptomatic infected people (*A*_*P*_), and removed people (*R*_*P*_) including recovered and death people. The birth rate and death rate of people were defined as *n*_*P*_ and *m*_*P*_. In this model, we set *Ʌ*_*P*_ *= n*_*P*_ × *N*_*P*_ where *N*_*P*_ refer to the total number of people. The incubation period and latent period of human infection was defined as 1/*ω*_*P*_ and 1/*ω’*_*P*_. The infectious period of *I*_*P*_ and *A*_*P*_ was defined as 1/*γ*_*P*_ and 1/*γ’*_*P*_. The proportion of asymptomatic infection was defined as *δ*_*P*_. The *S*_*P*_ will be infected through sufficient contact with *W* and *I*_*P*_, and the transmission rates were defined as *β*_*W*_ and *β*_*P*_, respectively. We also assumed that the transmissibility of *A*_*P*_ was *κ* times that of *I*_*P*_, where 0 ≤ *κ* ≤ 1.

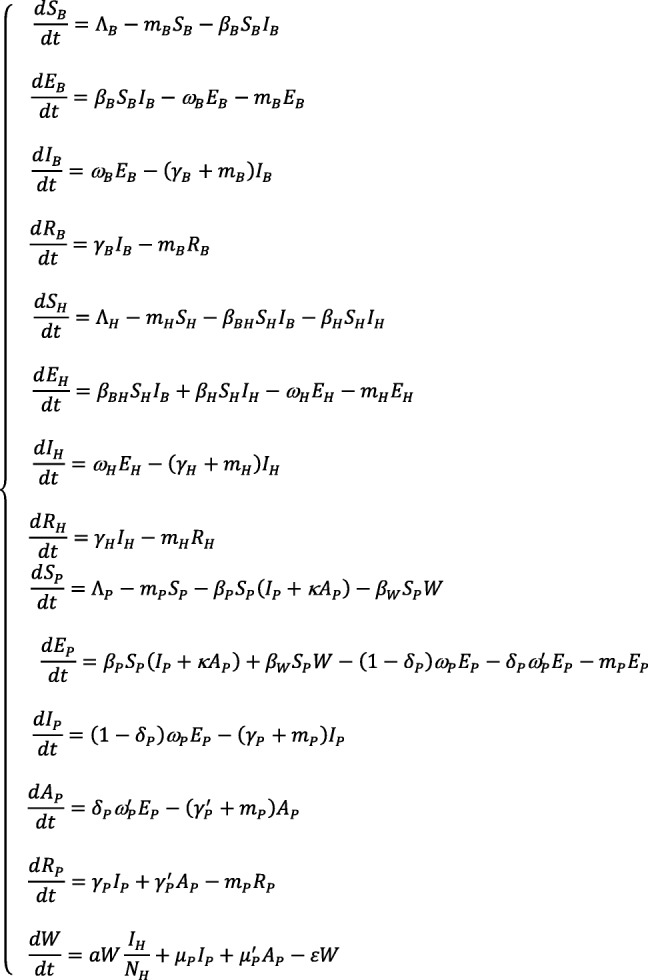



The parameters of the BHRP model were shown in Table 1.

**Table 1:** Definition of those parameters in the Bats-Hosts-Reservoir-People (BHRP) model

Parameter	Description
*n*_*B*_	The birth rate parameter of bats
*n*_*H*_	The birth rate parameter of hosts
*n*_*P*_	The birth rate parameter of people
*m*_*B*_	The death rate of bats
*m*_*H*_	The death rate of hosts
*m*_*P*_	The death rate of people
1/*ω*_*B*_	The incubation period of bats
1/*ω*_*H*_	The incubation period of hosts
1/*ω*_*P*_	The incubation period of people
1/*ω’*_*P*_	The latent period of people
1/*γ*_*B*_	The infectious period of bats
1/*γ*_*H*_	The infectious period of hosts
1/*γ*_*P*_	The infectious period of symptomatic infection of people
1/*γ’*_*P*_	The infectious period of asymptomatic infection of people
*β*_*B*_	The transmission rate from *I*_*B*_ to *S*_*B*_
*β*_*BH*_	The transmission rate from *I*_*B*_ to *S*_*H*_
*β*_*H*_	The transmission rate from *I*_*H*_ to *S*_*H*_
*β*_*P*_	The transmission rate from *I*_*P*_ to *S*_*P*_
*β*_*W*_	The transmission rate from *W* to *S*_*P*_
*a*	The retail purchases rate of the hosts in the market
*μ*_*P*_	The shedding coefficients from *I*_*P*_ to *W*
*μ’*_*P*_	The shedding coefficients from *A*_*P*_ to *W*
1/*ε*	The lifetime of the virus in *W*
*δ*_*P*_	The proportion of asymptomatic infection rate of people
*κ*	The multiple of the transmissibility of *A*_*P*_ to that of *I*_*P*_.

### The simplified reservoir-people transmission network model

We assumed that the SARS-CoV-2 might be imported to the seafood market in a short time. Therefore, we added the further assumptions as follows:

- The transmission network of Bats-Host was ignored.
- Based on our previous studies on simulating importation [13, 14], we set the initial value of *W* as following impulse function:
  $$ Importation= impulse\left(n,{t}_0,{t}_i\right) $$

In the function, *n*, *t*_0_ and *t*_*i*_ refer to imported volume of the SARS-CoV-2 to the market, start time of the simulation, and the interval of the importation.

Therefore, the BHRP model was simplified as RP model and is shown as follows:

$$ \left\{\kern0.5em \begin{array}{c}\frac{d{S}_P}{dt}={\varLambda}_P-{m}_P{S}_P-{\beta}_P{S}_P\left({I}_P+\upkappa {A}_P\right)-{\beta}_W{S}_PW\kern11em \\ {}\frac{d{E}_P}{dt}={\beta}_P{S}_P\left({I}_P+\upkappa {A}_P\right)+{\beta}_W{S}_PW-\left(1-{\delta}_P\right){\upomega}_P{E}_P-{\delta}_P{\upomega}_P^{\prime }{E}_P-{m}_P{E}_P\kern0.5em \\ {}\frac{d{I}_P}{dt}=\left(1-{\delta}_P\right){\upomega}_P{E}_P-\left({\gamma}_P+{m}_P\right){I}_P\kern16.5em \\ {}\frac{d{A}_P}{dt}={\delta}_P{\upomega}_P^{\prime }{E}_P-\left({\gamma}_P^{\prime }+{m}_P\right){A}_P\kern18.75em \\ {}\frac{d{R}_P}{dt}={\gamma}_P{I}_P+{\gamma}_P^{\prime }{A}_P-{m}_P{R}_P\kern20em \\ {}\frac{dW}{dt}={\mu}_P{I}_P+{\mu}_P^{\prime }{A}_P-\varepsilon W\kern20.5em \end{array}\right. $$

During the outbreak period, the natural birth rate and death rate in the population was in a relative low level. However, people would commonly travel into and out from Wuhan City mainly due to the Chinese New Year holiday. Therefore, *n*_*P*_ and *m*_*P*_ refer to the rate of people traveling into Wuhan City and traveling out from Wuhan City, respectively.

In the model, people and viruses have different dimensions. Based on our previous research [15], we therefore used the following sets to perform the normalization:

$$ {s}_P=\frac{S_P}{N_P},{e}_P=\frac{E_P}{N_P},{i}_P=\frac{I_P}{N_P}, {a}_P=\frac{A_P}{N_P},{r}_P=\frac{R_P}{N_P},w=\frac{\varepsilon W}{\mu_P{N}_P},\kern0.5em {\mu}_P^{\prime }=c{\mu}_P,\kern0.5em {b}_P={\beta}_P{N}_P,\mathrm{and}\ {b}_W=\frac{\mu_P{\beta}_W{N}_P}{\varepsilon .} $$

In the normalization, parameter *c* refers to the relative shedding coefficient of *A*_*P*_ compared to *I*_*P*_. The normalized RP model is changed as follows:

$$ \left\{\begin{array}{c}\frac{d{s}_P}{dt}={n}_P-{m}_P{s}_P-{b}_P{s}_P\left({i}_P+\upkappa {a}_P\right)-{b}_W{s}_Pw\\ {}\frac{d{e}_P}{dt}={b}_P{s}_P\left({i}_P+\upkappa {a}_P\right)+{b}_W{s}_Pw-\left(1-{\delta}_P\right){\upomega}_P{e}_P-{\delta}_P{\upomega}_P^{\prime }{e}_P-{m}_P{e}_P\\ {}\frac{d{i}_P}{dt}=\left(1-{\delta}_P\right){\upomega}_P{e}_P-\left({\gamma}_P+{m}_P\right){i}_P\\ {}\frac{d{a}_P}{dt}={\delta}_P{\upomega}_P^{\prime }{e}_P-\left({\gamma}_P^{\prime }+{m}_P\right){a}_P\kern26.5em \\ {}\frac{d{r}_P}{dt}={\gamma}_P{i}_P+{\gamma}_P^{\prime }{a}_P-{m}_P{r}_P\\ {}\frac{dw}{dt}=\varepsilon \left({i}_P+c{a}_P-w\right)\kern28.2em \end{array}\right. $$

### The transmissibility of the SARS-CoV-2 based on the RP model

In this study, we used the *R*_0_ to assess the transmissibility of the SARS-CoV-2. Commonly, *R*_0_ was defined as the expected number of secondary infections that result from introducing a single infected individual into an otherwise susceptible population [13, 16, 17]. If *R*_0_ > 1, the outbreak will occur. If *R*_0_ < 1, the outbreak will toward an end. In this study, *R*_0_ was deduced from the RP model by the next generation matrix approach [18].

### Parameter estimation

The parameters were estimated based on the following facts and assumptions:

- The mean incubation period was 5.2 days (95% confidence interval [*CI*]: 4.1–7.0) [3]. We set the same value (5.2 days) of the incubation period and the latent period in this study. Thus, *ω*_*P*_ = *ω’*_*P*_ = 0.1923.
- There is a mean 5-day delay from symptom onset to detection/hospitalization of a case (the cases detected in Thailand and Japan were hospitalized from 3 to 7 days after onset, respectively) [19–21]. The duration from illness onset to first medical visit for the 45 patients with illness onset before January 1 was estimated to have a mean of 5.8 days (95% *CI*: 4.3–7.5) [3]. In our model, we set the infectious period of the cases as 5.8 days. Therefore, *γ*_*P*_ = 0.1724.
- Since there was no data on the proportion of asymptomatic infection of the virus, we simulated the baseline value of proportion of 0.5 (*δ*_*P*_ = 0.5).
- Since there was no evidence about the transmissibility of asymptomatic infection, we assumed that the transmissibility of asymptomatic infection was 0.5 times that of symptomatic infection (*κ* = 0.5), which was the similar value as influenza [22]. We assumed that the relative shedding rate of *A*_*P*_ compared to *I*_*P*_ was 0.5. Thus, *c* = 0.5.
- Since 14 January, 2020, Wuhan City has strengthened the body temperature detection of passengers leaving Wuhan at airports, railway stations, long-distance bus stations and passenger terminals. As of January 17, a total of nearly 0.3 million people had been tested for body temperature [23]. In Wuhan, there are about 2.87 million mobile population [24]. We assumed that there was 0.1 million people moving out to Wuhan City per day since January 10, 2020, and we believe that this number would increase (mainly due to the winter vacation and the Chinese New Year holiday) until 24 January, 2020. This means that the 2.87 million would move out from Wuhan City in about 14 days. Therefore, we set the moving volume of 0.2 million per day in our model. Since the population of Wuhan was about 11 million at the end of 2018 [25], the rate of people traveling out from Wuhan City would be 0.018 (0.2/11) per day. However, we assumed that the normal population mobility before January 1 was 0.1 times as that after January 10. Therefore, we set the rate of people moving into and moving out from Wuhan City as 0.0018 per day (*n*_*P*_ = *m*_*P*_ = 0.0018).
- The parameters *b*_*P*_ and *b*_*W*_ were estimated by fitting the model with the collected data.
- At the beginning of the simulation, we assumed that the prevalence of the virus in the market was 1/100000.
- Since the SARS-CoV-2 is an RNA virus, we assumed that it could be died in the environment in a short time, but it could be stay for a longer time (10 days) in the unknown hosts in the market. We set *ε* = 0.1.

## Results

In this study, we assumed that the incubation period (1/*ω*_*P*_) was the same as latent period (1/*ω’*_*P*_) of human infection, thus *ω*_*P*_ = *ω’*_*P*_. Based on the equations of RP model, we can get the disease free equilibrium point as:

$$ \left(\frac{\varLambda_P}{m_P},0,0,0,0,0\right) $$

$$ F=\left[\begin{array}{cccc}0& {\beta}_P\frac{\varLambda_P}{m_P}& {\beta}_P\kappa \frac{\varLambda_P}{m_P}& {\beta}_W\frac{\varLambda_P}{m_P}\\ {}0& 0& 0& 0\\ {}0& 0& 0& 0\\ {}0& 0& 0& 0\end{array}\right],{V}^{-1}=\left[\begin{array}{cccc}\frac{1}{\omega_P+{m}_P}& 0& 0& 0\\ {}A& \frac{1}{\gamma_P+{m}_P}& 0& 0\\ {}B& 0& \frac{1}{\gamma_P^{\hbox{'}}+{m}_P}& 0\\ {}B& E& G& \frac{1}{\varepsilon}\end{array}\right] $$

In the matrix:

$$ A=\frac{\left(1-{\delta}_P\right){\upomega}_P}{\left({\upomega}_P+{m}_P\right)\left({\gamma}_P+{m}_P\right)} $$

$$ B=\frac{\delta_P{\upomega}_P}{\left({\upomega}_P+{m}_P\right)\left({\gamma}_p^{\prime }+{m}_P\right)} $$

$$ D=\frac{\left(1-{\delta}_P\right){\mu \upomega}_P}{\left({\upomega}_P+{m}_P\right)\left({\gamma}_P+{m}_P\right)\varepsilon }+\frac{\mu^{\prime }{\delta}_P{\upomega}_P}{\left({\upomega}_P+{m}_P\right)\left({\gamma}_p^{\prime }+{m}_P\right)\varepsilon } $$

$$ E=\frac{\mu }{\left({\gamma}_P+{m}_P\right)\varepsilon } $$

$$ G=\frac{\mu^{\prime }}{\left({\gamma}_p^{\prime }+{m}_P\right)\varepsilon } $$

By the next generation matrix approach, we can get the next generation matrix and *R*_0_ for the RP model:

$$ F{V}^{-1}=\left[\begin{array}{cccc}{\beta}_p\frac{\varLambda_P}{m_P}A+{\beta}_P\kappa \frac{\varLambda_P}{m_P}+{\beta}_W\frac{\varLambda_P}{m_P}D& \ast & \ast & \ast \\ {}0& 0& 0& 0\\ {}0& 0& 0& 0\\ {}0& 0& 0& 0\end{array}\right] $$

$$ {R}_0=\rho \left(F{V}^{-1}\right)={\beta}_P\frac{\varLambda_P}{m_P}\frac{\left(1-{\delta}_P\right){\omega}_P}{\left({\omega}_P+{m}_P\right)\left({\gamma}_P+{m}_P\right)}+{\beta}_P\kappa \frac{\varLambda_P}{m_P}\frac{\delta_P{\omega}_P}{\left({\omega}_P+{m}_P\right)\left({\gamma}_P^{\hbox{'}}+{m}_P\right)}+{\beta}_W\frac{\varLambda_P}{m_P}\frac{\left(1-{\delta}_P\right)\mu {\omega}_P}{\left({\omega}_P+{m}_P\right)\left({\gamma}_P+{m}_P\right)\varepsilon }+\beta W\frac{\varLambda_P}{m_P}\frac{\mu^{\hbox{'}}{\delta}_P{\omega}_P}{\left({\omega}_P+{m}_P\right)\left({\gamma}_P^{\hbox{'}}+{m}_P\right)\varepsilon } $$

The *R*_0_ of the normalized RP model is shown as follows:

$$ {R}_0={b}_p\frac{n_P}{m_p}\frac{\left(1-{\delta}_P\right){\omega}_P}{\left[\left(1-\delta p\right){\omega}_P+{\delta}_P{\omega}_P^{\hbox{'}}+{m}_P\right]\left({\gamma}_P+{m}_P\right)}+\kappa {b}_P\frac{n_P}{m_P}\frac{\delta_P{\omega}_P^{\hbox{'}}}{\left[\left(1-{\delta}_P\right){\omega}_P+{\delta}_P{\omega}_P^{\hbox{'}}+{m}_P\right]\left({\gamma}_P^{\hbox{'}}+{m}_P\right)}+{b}_W\frac{n_P}{m_P}\frac{\left(1-{\delta}_p\right){\omega}_p}{\left[\left(1-{\delta}_p\right){\omega}_P+{\delta}_P{\omega}_P^{\hbox{'}}+{m}_p\right]\left({\gamma}_P+{m}_P\right)}+{b}_W\frac{n_P}{m_P}\frac{c{\delta}_P{\omega}_P^{\hbox{'}}}{\left[\left(1-{\delta}_p\right){\omega}_P+{\delta}_P{\omega}_P^{\hbox{'}}+{m}_p\right]\left({\gamma}_P^{\hbox{'}}+{m}_P\right)} $$

**Fig. 2:**
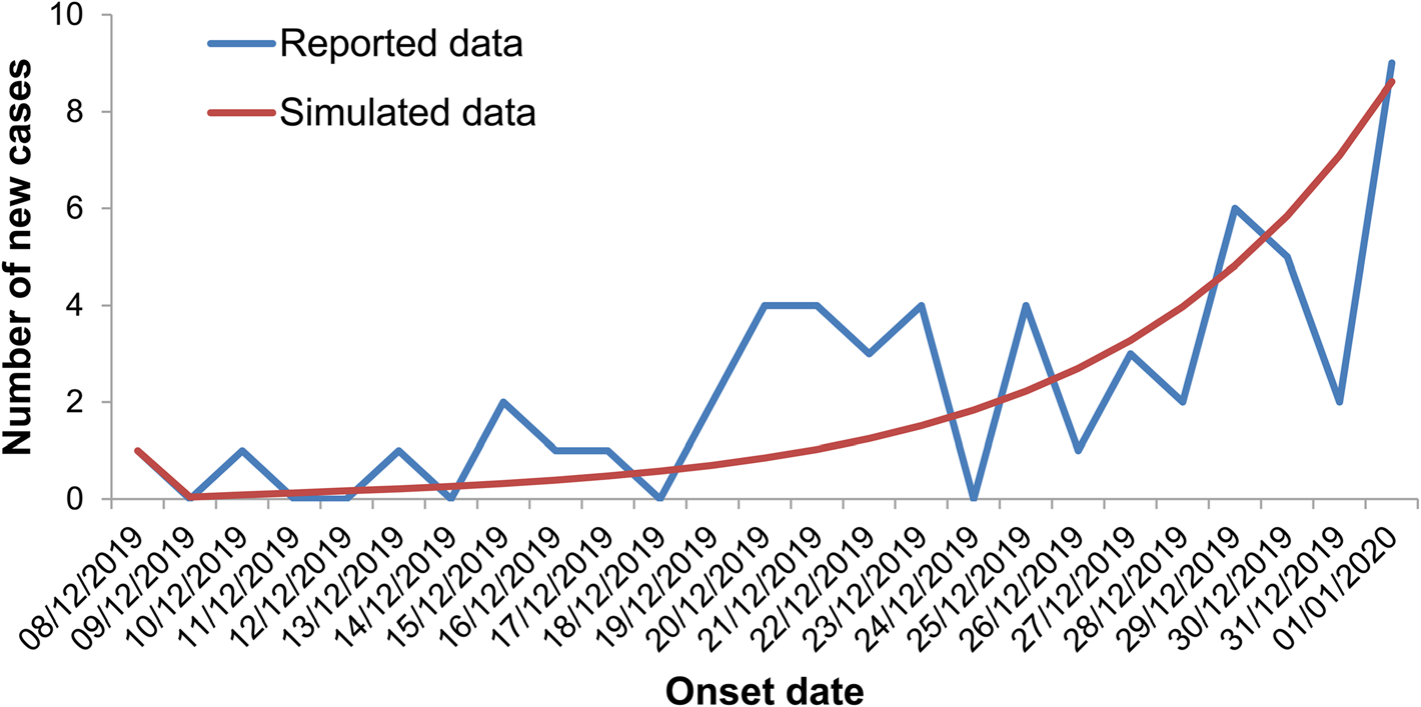
Curve fitting results of the RP model

Our modelling results showed that the normalized RP model fitted well to the reported SARS-CoV-2 cases data (*R*^2^ = 0.512, *P* < 0.001) (Fig. 2). The value of *R*_0_ was estimated of 2.30 from reservoir to person, and from person to person and 3.58 from person to person which means that the expected number of secondary infections that result from introducing a single infected individual into an otherwise susceptible population was 3.58.

## Discussion

In this study, we developed RP transmission model, which considering the routes from reservoir to person and from person to person of SARS-CoV-2 respectively. We used the models to fit the reported data in Wuhan City, China from published literature [3]. The simulation results showed that the *R*_0_ of SARS-CoV-2 was 3.58 from person to person. There was a research showed that the *R*_0_ of SARS-CoV-2 was 2.68 (95% *CI*: 2.47–2.86) [8]. Another research showed that the *R*_0_ of SARS-CoV-2 was 2.2 (95% *CI*: 1.4–3.9) [3]. The different values might be due to the different methods. The methods which Li et al. employed were based on the epidemic growth rate of the epidemic curve and the serial interval [3]. Our previous study showed that several methods could be used to calculate the *R*_0_ based on the epidemic growth rate of the epidemic curve and the serial interval, and different methods might result in different values of *R*_0_ [26]. Our results also showed that the *R*_0_ of SARS-CoV-2 was 2.30 from reservoir to person which was lower than that of person to person. This means that the transmission route was mainly from person to person rather than from reservoir to person in the early stage of the transmission in Wuhan City. However, this result was based on the limited data from a published literature, and it might not show the real situation at the early stage of the transmission.

Researches showed that the *R*_0_ of severe acute respiratory syndrome (SARS) was about 2.7–3.4 or 2–4 in Hong Kong, China [27, 28]. Another research found that the *R*_0_ of SARS was about 2.1 in Hong Kong, China, 2.7 in Singapore, and 3.8 in Beijing, China [29]. Therefore, we believe that the commonly acceptable average value of the *R*_0_ of SARS might be 2.9 [30]. The transmissibility of the Middle East respiratory syndrome (MERS) is much lower than SARS. The reported value of the *R*_0_ of MERS was about 0.8–1.3 [31], with the inter-human transmissibility of the disease was about 0.6 or 0.9 in Middle East countries [32]. However, MERS had a high transmissibility in the outbreak in the Republic of Korea with the *R*_0_ of 2.5–7.2 [33, 34]. Therefore, the transmissibility of SARS-CoV-2 might be higher than MERS in the Middle East countries, similar to SARS, but lower than MERS transmitted in the Republic of Korea.

To contain the transmission of the virus, it is important to decrease *R*_0_. According to the equation of *R*_0_ deduced from the simplified RP model, *R*_0_ is related to many parameters. The mainly parameters which could be changed were *b*_*P*_, *b*_*W*_, and *γ*. Interventions such as wearing masks and increasing social distance could decrease the *b*_*P*_, the intervention that close the seafood market could decrease the *b*_*W*_, and shorten the duration form symptoms onset to be diagnosed could decrease 1/*γ*. All these interventions could decrease the effective reproduction number and finally be helpful to control the transmission.

Since there are too many parameters in our model, several limitations exist in this study. Firstly, we did not use the detailed data of the SARS-CoV-2 to perform the estimation instead of using the data from literatures [3]. We simulated the natural history of the infection that the proportion of asymptomatic infection was 50%, and the transmissibility of asymptomatic infection was half of that of symptomatic infection, which were different to those of MERS and SARS. It is known that the proportion of asymptomatic infection of MERS and SARS was lower than 10%. Secondly, the parameters of population mobility were not from an accurate dataset. Thirdly, since there was no data of the initial prevalence of the virus in the seafood market, we assumed the initial value of 1/100 000. This assumption might lead to the simulation been under- or over-estimated. In addition, since we did not consider the changing rate of the individual’s activity (such as wearing masks, increasing social distance, and not to travel to Wuhan City), the estimation of importation of the virus might not be correct. All these limitations will lead to the uncertainty of our results. Therefore, the accuracy and the validity of the estimation would be better if the models fit the first-hand data on the population mobility and the data on the natural history, the epidemiological characteristics, and the transmission mechanism of the virus.

## Conclusions

By calculating the published data, our model showed that the transmissibility of SARS-CoV-2 might be higher than MERS in the Middle East countries, similar to SARS, but lower than MERS in the Republic of Korea. Since the objective of this study was to provide a mathematical model for calculating the transmissibility of SARS-CoV-2, the *R*_0_ was estimated based on limited data which published in a literature. More data were needed to estimate the transmissibility accurately.

## Data Availability

Not applicable.

## Abbreviations

2019-nCoV: 2019 novel coronavirus
BHRP: Bats-Hosts-Reservoir-People
*R*_0_: Basic reproduction number
RP: Reservoir-People
SARS-CoV-2: Severe acute respiratory syndrome coronavirus 2
WHO: World Health Organization
